# Assessment of the Promoting Resilience in Stress Management Intervention for Adolescent and Young Adult Survivors of Cancer at 2 Years: Secondary Analysis of a Randomized Clinical Trial

**DOI:** 10.1001/jamanetworkopen.2021.36039

**Published:** 2021-11-24

**Authors:** Abby R. Rosenberg, Chuan Zhou, Miranda C. Bradford, John M. Salsman, Katie Sexton, Alison O’Daffer, Joyce P. Yi-Frazier

**Affiliations:** 1 Palliative Care and Resilience Lab, Seattle Children’s Research Institute, Seattle, Washington; 2 Division of Hematology/Oncology, Department of Pediatrics, University of Washington School of Medicine, Seattle; 3 Cambia Palliative Care Center of Excellence, University of Washington, Seattle; 4 Center for Child Health, Behavior, and Development, Seattle Children’s Research Institute, Seattle, Washington; 5 Division of General Pediatrics, Department of Pediatrics, University of Washington School of Medicine, Seattle; 6 Biostatistics, Epidemiology, and Analytics in Research Program, Seattle Children’s Research Institute, Seattle, Washington; 7 Wake Forest School of Medicine and Wake Forest Baptist Comprehensive Cancer Center, Winston Salem, North Carolina; 8 Department of Medical Education, University of Washington School of Medicine, Seattle

## Abstract

**Importance:**

Adolescents and young adults (AYAs) with cancer have a high risk of poor psychosocial outcomes. The Promoting Resilience in Stress Management (PRISM) intervention is one of few psychosocial interventions targeting younger people with cancer that has demonstrated efficacy in a randomized clinical trial.

**Objective:**

To explore 2-year trajectories of patient-reported well-being among AYA cancer survivors. The hypothesis was that AYAs who initially responded to PRISM would report sustained positive changes.

**Design, Setting, and Participants:**

This secondary analysis of data from a single-center, parallel, phase 2 randomized clinical trial was conducted from January 2015 to October 2016. Eligible participants were English-speaking AYAs between ages 13 and 25 years with cancer treated at a single, quaternary children’s hospital in the US. Participants were randomly assigned (1:1) to PRISM or usual care. Data were analyzed between March 2021 and June 2021.

**Intervention:**

PRISM is a brief, skills-based coaching program targeting 4 resilience resources (stress management, goal setting, cognitive reframing, and meaning making).

**Main Outcomes and Measures:**

Patient-reported cancer-related quality of life (QoL) (PedsQL scale), hope (Hope scale), resilience (Connor-Davidson Resilience scale), and psychological distress (Kessler-6 scale) were collected at baseline, 6, 12, and 24 months. Data from AYAs who remained alive at 24 months were analyzed. Improvement was defined as a positive change in instrument scores between baseline and 6 months (ie, the end of the PRISM program) and used linear mixed effects regression to assess changes over time.

**Results:**

A total of 57 AYAs were included in analysis at 24 months (62% of 92 randomized). Twenty-six participants (46%) were female; 36 (63%) were between ages 13 and 17 years; and 20 (35%) identified as part of a racial or ethnic minority group (4 [7%] Asian, 11 [19%] Hispanic or Latino, 16 [28%] mixed race or other). PRISM was associated with improved QoL, hope, resilience, and distress between baseline and 6 months, and these associations were sustained for QoL over 12 months (β = 7.4; 95% CI, 0.8 to 14; *P* = .03) and hope throughout the study period (12 months: β = 6.2; 95% CI, 2.7 to 9.6; *P* < .001; 24 months: β = 4.6; 95% CI, 1 to 8.3; *P* = .01). Of those whose scores improved between baseline and 6 months, PRISM participants more commonly reported sustained improvements in QoL (16 of 21 participants [76%] vs 8 of 17 [47%]), hope (14 of 24 participants [58%] vs 6 of 14 [43%]), resilience (9 of 18 participants [50%] vs 4 of 14 [29%]), and distress (14 of 19 participants [74%] vs 6 of 19 [32%]) at 24 months compared with usual care.

**Conclusions and Relevance:**

These results suggest that PRISM was associated with sustained improvements in psychosocial outcomes as reported by adolescents and young adults. Those who responded to PRISM in the first 6 months reported continued well-being 2 years later.

**Trial Registration:**

ClinicalTrials.gov Identifier: NCT02340884

## Key Points

**Question:**

Is the Promoting Resilience In Stress Management (PRISM) psychosocial intervention associated with sustained well-being among adolescent and young adult cancer survivors?

**Findings:**

In this secondary analysis of available 2-year long-term follow-up data on 57 participants from a phase 2 clinical randomized trial, adolescents and young adults who received PRISM reported sustained improvements in patient-reported cancer-related quality of life and hope compared with those who received usual care.

**Meaning:**

The findings of this study suggest that positive psychology interventions such as PRISM may bolster durable well-being among adolescent and young adult survivors of cancer.

## Teaser

This secondary analysis of long-term follow-up data from a randomized clinical trial examines the effectiveness of the Promoting Resilience In Stress Management intervention for the psychological well-being of adolescents and young adults who have survived cancer.

## Introduction

The poor psychosocial outcomes of adolescent and early young adult (AYA; defined as ages 13 to 25 years) survivors of cancer are well-established. Compared with age-matched peers, AYAs who survive cancer report higher psychological distress and fewer positive health beliefs than their younger pediatric or older adult counterparts.^1,2,3,4^ Impaired physical, social, and emotional health during and after cancer therapy are common.^5,6,7,8,9,10,11^ Reasons for these disparate experiences include the fact that cancer among AYAs disrupts normal developmental processes like the establishment of personal, social, and sexual identity and the pursuit of educational and vocational goals.^6,12,13^ Moreover, AYAs diagnosed with cancer may not have developed the positive psychological life skills necessary to cope with the stressors of cancer therapy and early survivorship.

Despite suggestions by national organizations to address these disparities and needs, evidence-based, AYA-specific psychosocial interventions are rare.^13,14,15,16^ The few that have been empirically evaluated promote survivorship knowledge, lifestyle change, and physical fitness.^17,18^ While these may indirectly support AYA quality of life (QoL), no interventions have successfully reduced distress or durably improved AYA well-being. The latter point is important because well-being is complex; it is defined as holistic life satisfaction, the presence of positive affect, and the lack of negative affect.^19^ Perhaps our stalled progress in improving long-term AYA psychosocial outcomes is because we have not successfully targeted all 3 aspects of well-being.

With this in mind, we created the Promoting Resilience in Stress Management (PRISM) intervention.^20^ PRISM is one of the first positive psychology interventions designed specifically for AYAs with cancer, and the only intervention to date with established efficacy in a randomized clinical trial (RCT). A phase 2 RCT comparing PRISM with usual care among AYAs with newly diagnosed or newly recurrent cancer suggested the program improved patient-reported cancer-related QoL, hope, and resilience while reducing psychological distress 6 months after enrollment.^21,22^ Indeed, PRISM was designed with AYA-patient partnership to be a brief, skills-based program targeting positive psychological resources during periods of acute stress (ie, early after diagnosis, in the setting of progressive disease, or early in the transition to survivorship care). Our hope was that these skills would, in turn, support long-lasting well-being.

The purpose of the present analysis was to explore 2-year trajectories of key components of patient-reported well-being (cancer-related QoL, hope, resilience, and psychological distress) among AYAs who did and did not receive PRISM. We anticipated that AYAs who responded to the initial intervention would report sustained positive changes.

## Methods

This study is a secondary analysis of data from a single-institution, phase 2, parallel RCT with 1:1 randomization. Study methods and primary results after 6 months have been presented previously.^21,22,23,24,25,26^ For this analysis, we followed the Consolidated Standards of Reporting Trials (CONSORT) reporting guideline for randomized trials to report follow-up data from AYAs who remained alive and on-study 2 years later.

### Design, Setting, and Participants

AYAs were eligible to enroll if they were: (1) between ages 13 and 25 years; (2) fluent in spoken and written English, including as a second language; (3) diagnosed with either new or progressive malignant neoplasm treated with systemic chemotherapy; and (4) deemed by clinical staff and/or caregivers to be cognitively able to participate in the intervention. All participants were treated at a large quaternary children’s hospital (Seattle Children’s Hospital). They enrolled between January 2015 and October 2016. The hospital institutional review board approved the study (protocol available as Supplement 1).

### Recruitment, Enrollment, and Randomization

Study staff identified potential participants through clinic rosters, inpatient census reports, and tumor board registries, and approached them via outpatient clinics or inpatient wards. Following detailed discussions about the study purpose, risks, and benefits, adolescents (ages 13 to 17 years) provided written assent and their parents or caregivers provided written consent. Young adults (ages 18 to 25 years) provided written consent.

**Figure 1.**
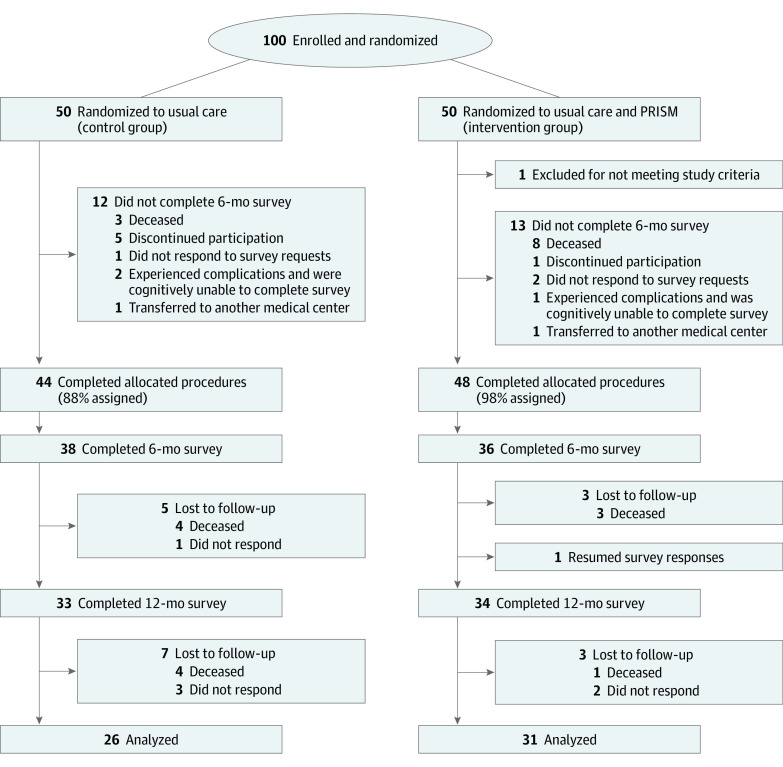
CONSORT Diagram of Study Randomization and Retention From Enrollment Through 2 Years Follow-up PRISM indicates Promoting Resilience in Stress Management intervention.

We enrolled 100 consecutive AYAs who provided informed consent (Figure 1). This sample size was based on realistic expectations for enrollment at our single center, our primary outcome (ie, patient-reported 10-item Connor-Davidson Resilience Scale [CDRISC-10] scores 6-month postenrollment), and preliminary data suggesting scores were normally distributed with a mean (SD) score of 31 (5.3).^21^ We defined the minimal clinically important difference (MCID; the smallest difference in scores where patients report a clinical benefit) a priori as half the SD^27^ and anticipated 10% attrition; 90 participants (45 per arm) provided 80% power with 2-sided α = .05 to detect the MCID in patient-reported resilience at 6 months.

Enrolled participants were randomly assigned to usual care (control) alone or usual care plus PRISM (intervention) in a 1:1 ratio. The study statistician (C.Z.) constructed the randomization algorithm using permuted blocks with varying sizes, stratified by age (13 to 17 years vs 18 to 25 years). Staff were masked to the randomization scheme until after enrollment.

### Psychosocial Usual Care

All participants received psychosocial usual care. This included an assigned social worker who conducted comprehensive psychosocial assessments upon initiation of care and provided ad hoc support thereafter. Common ad hoc services included financial, housing, and concrete supportive care for families, plus intermittent mental health support for AYAs. Medical staff referred patients to additional subspecialty mental health support based on patient or family request or observed psychological distress.

### The PRISM Intervention

PRISM was founded on stress-and-coping and resilience theories and prior patient-centered research suggesting AYAs with cancer rely on 4 reproducible “resilience resources” to navigate their cancer experience.^20,28,29^ PRISM teaches stress management (relaxation and mindfulness skills, including deep breathing and becoming aware of stressors without judgement), goal setting (setting SMART [Specific, Measurable, Actionable, Realistic, Time-dependent] goals, enabling observable progress toward an achievable hope), positive reframing (recognizing negative emotions and demoralizing self-talk and reframing perceptions realistically and/or positively), and benefit finding (finding meaning and gratitude in difficult situations, including cancer). In this study, each of the 4 sessions was taught 1-on-1 by a certified coach and lasted between 20 and 50 minutes. Sessions were delivered approximately every other week. Between sessions, participants received worksheets to further develop skills.

PRISM was administered by trained, bachelors-level nonclinical coaches.^20^ All received 8 hours or more of standardized training including roleplaying and mock sessions. AYAs were assigned a single coach for the whole program; coaches administered each session in a private patient room during inpatient hospital stays or before or after outpatient clinic visits.

### Study Instruments

We queried demographic variables via surveys and collected cancer-related data from participants’ medical record. All participants were invited to complete a survey consisting of AYA age-validated instruments upon enrollment, and then 6, 12, and 24 months later.

The survey included 4 patient-reported variables measuring patient well-being: cancer-related QoL, hope, resilience, and psychological distress. Cancer-related QoL was measured using the Pediatric Quality of Life (PedsQL) cancer module, which assesses cancer-related symptoms, worries, cognition, and communication.^30,31^ Total scores range from 0 to 100, with higher scores representing better quality of life. Hopeful patterns of thought were measured with the Hope Scale, which examines “the overall perception that one’s goals can be met.”^32^ Items distinguish between perceived ability to generate a route to one’s goals (referred to as “pathway”) and ability to initiate and maintain actions to achieve those goals (“agency”). It is scored on an 8-point Likert scale (range, 8-64); higher scores imply greater levels of hopeful thought. Resilience was measured with the 10-item Connor Davidson Resilience Scale (CDRISC-10), which assesses self-perceived resilience by querying how an individual handles adversity.^33^ Items are scored on a 5-point Likert scale; total scores range from 0 to 40 with higher scores reflecting greater resilience. Finally, psychological distress was measured using the Kessler-6 psychological distress scale measures level of global psychological distress.^34,35^ Scores range from 0 to 24 points; higher scores reflect greater distress.

### Procedures

Following randomization, staff notified each AYA of their assignment and delivered baseline surveys. Thereafter, participants received in-person, phone, and/or email reminders 7 days prior to each session (PRISM intervention arm) or survey due date (all participants). Following completion of the primary end point (ie, 6 months), staff monitored the medical records of each AYA and recorded major events including relocation of care, relapse, or death. Surveys were requested at 12 and 24 months with no interval contact between staff and participants. For participants known to be alive, when surveys were not returned, staff contacted participants once weekly for 3 weeks. Participants received a $25 gift card upon completion of baseline, and $50 for surveys thereafter.

### Statistical Analyses

The objective of this analysis was to explore 2-year trajectories of AYA-reported outcomes. We conducted an intention-to-treat analysis and included data from all patients who completed baseline surveys and remained alive at 24 months. Because the majority of missing data was due to patient death, missingness was deemed not at random. We presumed that experiences of AYAs who survived their cancer for 24 months were unlike those of AYAs who died within that timeframe. Existing statistical methods for data missing not at random are complex and rely on strong assumptions, therefore we based our analysis on observed data only.

We summarized demographic characteristics and instrument scores for all participants at each time point. To explore the pattern of continued change in well-being after PRISM, we defined a participant as improved if a positive change in instrument scores between baseline and 6 months (ie, the end of active PRISM intervention) was observed. We then calculated the percentages of participants whose scores remained improved from baseline at later time points. Finally, we performed a difference-in-differences analysis using linear mixed effects regression models to assess how changes over time in well-being differed between PRISM and usual care arms. This was done by including a study arm by time interaction term in models. Patient-specific random intercept was included to account for clustering due to repeated assessments. The estimation was based on restricted maximum likelihood and hypothesis testing was with Kenward-Roger approach.^36,37^ All analyses were conducted the R statistical software version 4.1.0 (R Project for Statistical Computing).^38^

## Results

Of the 57 AYAs who were alive and on-study at 24 months, 26 (46%) were female, 36 (63%) were between ages 13 to 17 years, 20 (35%) identified as part of a racial or ethnic minority group (4 [7%] Asian, 11 [19%] Hispanic or Latino, 16 [28%] mixed race or other), and 5 (9%) spoke English as a second language (Table 1). The most common diagnoses (37 [65%]) were leukemia or lymphoma.

**Table 1.** Participant Characteristics at Time of Enrollment, 6 Months, 12 Months, and 24 Months

Characteristics	Participants, No. (%)							
	Baseline		6 mo		12 mo		2 y	
	PRISM (n = 48)	Usual care (n = 44)	PRISM (n = 36)	Usual care (n = 38)	PRISM (n = 34)	Usual care (n = 33)	PRISM (n = 31)	Usual care (n = 26)
Sex								
Female	16 (33)	24 (55)	12 (33)	21 (55)	11 (32)	16 (48)	12 (39)	14 (54)
Male	32 (67)	20 (45)	24 (67)	17 (45)	23 (68)	17 (52)	19 (61)	12 (46)
Age at enrollment, y								
12-17	35 (73)	32 (73)	27 (75)	26 (68)	26 (76)	22 (67)	23 (74)	16 (62)
18-25	13 (27)	12 (27)	9 (25)	12 (32)	8 (24)	11 (33)	8 (26)	10 (38)
Age, mean (SD), y	17 (3)	16 (3)	17 (3)	17 (3)	17 (2)	18 (3)	18 (3)	19 (4)
Race^a^								
Asian	6 (3)	3 (7)	2 (6)	3 (7)	2 (6)	2 (6)	2 (6)	2 (8)
Black or African American	2 (4)	0	1 (3)	0	0	0	0	0
Other	5 (10)	12 (27)	3 (8)	10 (26)	3 (9)	10 (30)	3 (10)	7 (27)
Mixed race	4 (8)	4 (9)	3 (8)	4 (11)	3 (9)	3 (9)	3 (10)	3 (12)
White	34 (71)	25 (57)	27 (75)	21 (55)	26 (76)	18 (55)	23 (74)	14 (54)
Hispanic or Latino	5 (10)	17 (39)	3 (8)	13 (34)	2 (6)	13 (39)	2 (6)	9 (35)
First language other than English	1 (2)	10 (23)	3 (8)	9 (24)	1 (3)	6 (19)	1 (3)	4 (16)
Leukemia/lymphoma	31 (65)	29 (66)	23 (66)	25 (66)	22 (69)	22 (69)	19 (63)	18 (72)
CNS	3 (6)	4 (9)	2 (6)	3 (8)	1 (3)	3 (9)	2 (7)	2 (8)
Non-CNS solid tumor	14 (29)	11 (25)	8 (23)	10 (26)	7 (22)	7 (22)	4 (13)	5 (20)
Diagnosed cancer at enrollment								
Newly	38 (79)	30 (68)	30 (83)	27 (71)	28 (82)	25 (76)	26 (84)	20 (77)
Advanced	10 (21)	14 (32)	6 (17)	11 (29)	6 (18)	8 (24)	5 (16)	6 (23)
Recurrence between baseline and later survey date	NA	NA	1 (3)	3 (8)	2 (6)	1 (3)	2 (6)	3 (12)

Abbreviations: CNS, central nervous system; NA, not applicable; PRISM, Promoting Resilience in Stress Management.

^a^ Participants were asked to select a single race category; “other” was self-selected by participants and did not include additional participant-reported details.

As described previously, we enrolled and randomized 100 of 130 eligible AYAs (77%) during the enrollment period (Figure 1).^21^ Following randomization, 1 AYA was determined ineligible (did not read English) and 7 discontinued participation prior to baseline surveys, leaving 92 for planned analyses (44 usual care, 48 PRISM). Over the subsequent 24 months, 35 AYAs discontinued study participation. Of these, 24 (26% of total sample, 69% of attrition) discontinued due to critical illness (2 [2%] in total sample) or death (22 [24%] in total sample). Two participants (2%) transferred care elsewhere, and 9 (10%) declined to return surveys and were thus determined to have discontinued passively. Attrition for all reasons was similar in each arm.

Across all domains of well-being, including after adjustment for age, sex, race and ethnicity, and English as primary or secondary language, PRISM recipients improved between baseline and 6 months (eTable in Supplement 2).^21,22^ Scores at 6 months were similar between subgroups of AYAs who did and did not subsequently experience recurrence or death.

**Figure 2.**
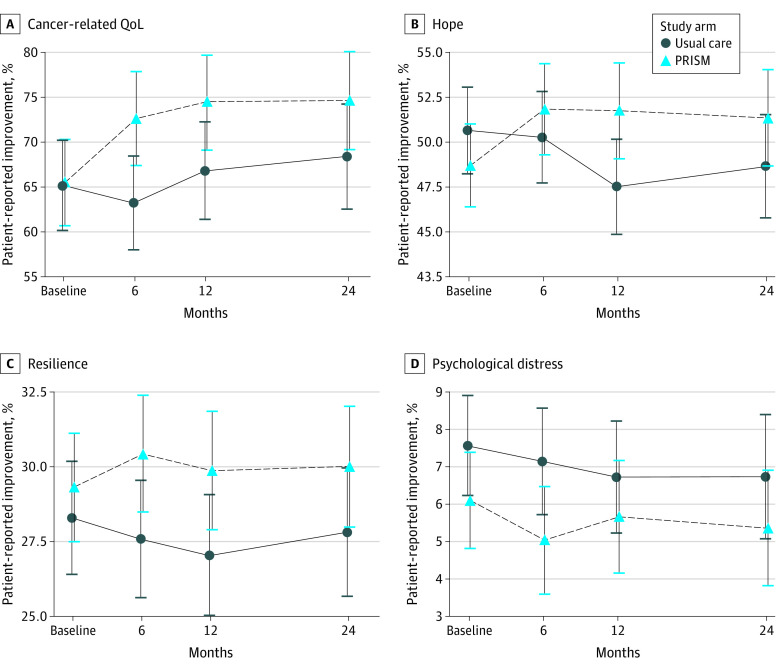
Trajectories of Patient-Reported Outcomes Among Patients Who Were Alive at 24 Months Points represent mean scores and error bars 95% CI at each time point of baseline (enrollment), 6, 12, and 24 months thereafter. PRISM indicates Promoting Resilience in Stress Management intervention; QoL, quality of life.

For cancer-related quality of life, PRISM was associated with an improvement from baseline to 6 months (β = 9.1; 95% CI, 2.8 to 15.4; *P* = .01) and 12 months (β = 7.4; 95% CI, 0.8 to 14; *P* = .03) but not at 2 years (β = 5.9; 95% CI, −1.1 to 12.9; *P* = .10) (Figure 2). There was significant group-by-time interaction, suggesting the change in scores over time differed significantly between groups.

**Figure 3.**
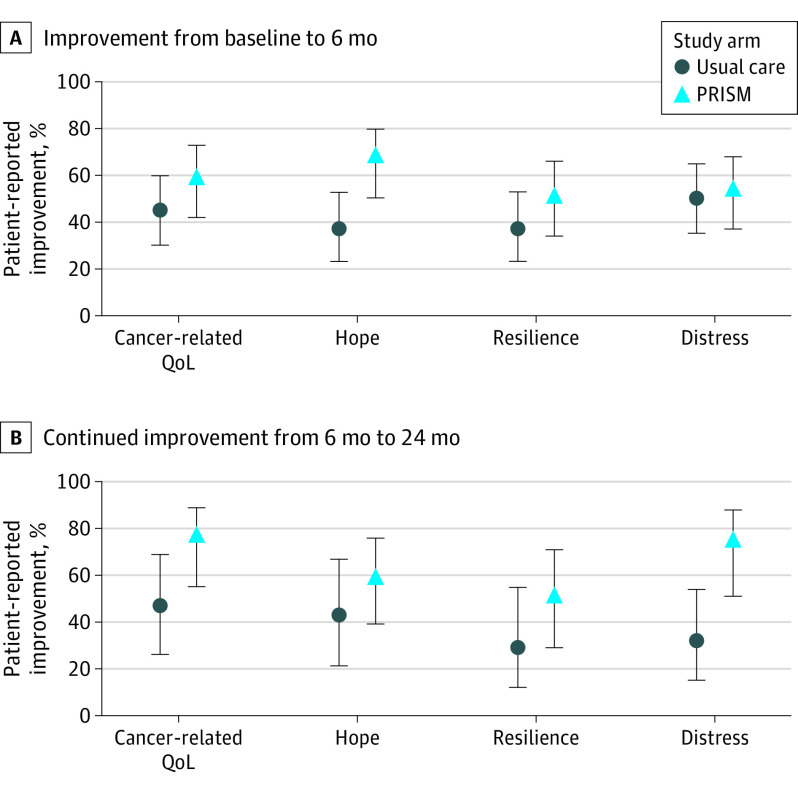
Percentage of Adolescents and Young Adults in Each Study Group Who Improved Between Baseline and 6 Months and Were Alive and Who Remained Improved at 24 Months PRISM indicates Promoting Resilience in Stress Management; QoL, quality of life.

For hope, PRISM-participants reported significant improvements between baseline and 6-month (β = 3.5; 95% CI, 0.3 to 6.8; *P* = .04) and their scores remained higher than baseline at 12 months (β = 6.2; 95% CI, 2.7 to 9.6; *P* < .001), and at 2 years (β = 4.6; 95% CI, 1.0 to 8.3; *P* = .01) (Figure 2). There was significant group-by-time interaction. For resilience, PRISM-participants did not report significantly more improvement from baseline at 6 months (β = 1.8; 95% CI, −0.4 to 4.0; *P* = .10). Their scores were not statistically significantly higher than usual care–recipient improvements from baseline at 12 months (β = 1.8, 95% CI −0.5-4, *P* = .12) or 24 months (β = 1.2, 95% CI −1.2 to 3.6, *P* = .34) (Figure 2). There was no significant group-by-time interaction. For psychological distress, there were no differences between groups with respect to sustained differences in scores from baseline to 6 months (β = −0.7; 95% CI, −2.7 to 1.4; *P* = .54), 12 months (β = 0.4; 95% CI, −1.8 to 2.6; *P* = .72), or 24 months (β = 0.1; 95% CI, −2.2 to 2.4; *P* = .94) (Figure 2). Of the AYAs who improved in either arm, 29% to 47% of usual care participants vs 50% to 76% of PRISM participants maintained improvement status in patient reported outcomes from 6 months to 24 months (Table 2; Figure 3).

**Table 2.** 24-Month Outcomes for Participants With Improved Self-reported Outcome Scores at 6 Months

Patient-reported outcome	Participants, No. (%)							
	Cancer-specific QoL		Hope		Resilience		Psychological distress	
	PRISM	Usual care	PRISM	Usual care	PRISM	Usual care	PRISM	Usual care
Improved at 6 mo	21/36 (58)	17/38 (45)	24/36 (67)	14/38 (37)	18/36 (50)	14/38 (37)	19/36 (53)	19/38 (50)
Still improved at 24 mo								
Yes	16/21 (76)	8/17 (47)	14/24 (58)	6/14 (43)	9/18 (50)	4/14 (29)	14/19 (74)	6/19 (32)
No	2/21 (10)	2/17 (12)	6/24 (25)	4/14 (29)	5/18 (28)	5/14 (36)	4/19 (21)	3/19 (16)
Missing	3/21 (14)	7/17 (41)	4/24 (17)	4/14 (29)	4/18 (22)	5/14 (36)	1/19 (5)	10/19 (52)

Abbreviations: PRISM, Promoting Resilience in Stress Management; QoL, quality of life.

## Discussion

In this exploratory analysis of long-term follow-up data from the PRISM phase 2 RCT, up to three-quarters of AYAs who received PRISM reported sustained improvements from baseline in well-being domains. Fewer than half of those who received usual care alone noted the same. Moreover, AYAs who immediately responded to PRISM (ie, with improved scores between baseline and 6 months) reported greater degrees of sustained improvements in QoL and hope. We saw no difference between groups with respect to durable differences in distress, although those whose distress went down with PRISM tended to remain distress-free thereafter. Together, these findings suggest that: (1) psychosocial interventions targeting positive psychological outcomes have benefit; (2) immediate response to such programs may give an accurate estimate of long-term well-being; and (3) positive psychology interventions may not alleviate the later psychological distress.

The need for targeted psychosocial interventions for AYAs with cancer is well-established.^1,2,3,4^ Patients in this age group have well-described risks of long-term poor physical and emotional health outcomes.^5,6,7,8,9,10,11^ However, few interventions to date have successfully minimized risks of long-term distress and fewer still have promoted durable positive outcomes.^14^ Perhaps the rarity of success in this age group is because we have selected incomplete targets. While it seems intuitive to focus explicitly on minimizing psychological distress, data regarding the utility of this approach are mixed. While some long-term pediatric survivorship data suggest similar rates of psychopathology among survivors and age-matched controls, most suggest this population has substantially higher risks of ongoing distress and poor mental health.^39,40,41,42,43,44,45,46,47,48^ Similarly, only a subset of people who experience traumatic stressors as diverse as death of a loved one, natural disaster, or disease have lasting functional impairments.^49,50^ In other words, long-term distress seems neither predictable nor avoidable. Furthermore, psychological distress is more commonly impacted by specific symptoms (ie, worry, strain, and feelings of worthlessness) and less affected by positive affect or overall quality of life.^51^ These findings lead us to ask if, instead of “How to avoid distress?” the important question for treating AYA cancer survivors is, “How to promote overall well-being in spite of increased risks for distress?”

Well-being is broadly defined as 3 separate and interrelated constructs: life satisfaction or quality of life, the presence of positive affect, and the absence of negative affect.^52^ Each component is clinically meaningful; positive outlook, benefit-finding, and hope during cancer therapy are protective—and significant distress is a risk factor—for long-term maladjustment and poor function.^40,53^ Evidence suggests that brief interventions targeting patient well-being during times of acute stress may be the most effective.^40^ While psychological distress may be transient and, at times, normal or unavoidable, learned positive psychological resources endure and buffer the effects of recurrent stress or distress.^19,54^

PRISM was designed with these 3 perspectives in mind. Our prior data suggest it bolsters all 3 aspects of well-being in the short-term. In the long-term, patients who received PRISM seem more likely to report higher quality of life and greater hope. They are just as likely as their peers to report an absence of distress. Taken together, PRISM may support overall well-being in an enduring manner.

### Limitations

This study has several important limitations to note. First, our small sample was mostly White, English-speaking adolescents from a single center. PRISM is also a resource-intensive program that requires a 1:1 coach. Our findings may not be generalizable to diverse populations in other settings. Second, we lacked power to discern differences in outcomes based on age, gender, or other medical, psychosocial, or sociodemographic characteristics. Third, the heterogeneity of the sample with respect to cancer-type and new vs recurrent disease may have contributed to variable response and attrition. For example, there were more AYAs with newly diagnosed (vs recurrent) cancer assigned to PRISM. This may have contributed to PRISM’s superior outcomes. Also, while 6-month instrument scores were not associated with later recurrence or death, sustained improvement in outcomes may have been less likely among AYAs who experienced recurrence after 6 months. Indeed, a substantial proportion of our initial sample died in the 24 months of observation; the present sample may represent a healthier (and therefore biased) population who experienced fewer medical complications. Finally, we did not collect data regarding other stressors or life experiences such as transitions of care, school and/or employment, mental health challenges, social supports, or other elements of AYA QoL, or regarding use of ongoing medical and psychosocial services after completion of the study. Such information is critical because all these elements contribute to overall AYA well-being. Because distress, in particular, is associated with transient experiences and symptoms,^51^ we cannot determine what contributed to positive or negative trajectories.

Nevertheless, this exploratory study raises important considerations for AYA clinical care and future research. First, our findings underscore the idea that supporting patients during times of acute stress is key. Even if the prevalence of long-term distress is similar among AYAs who received PRISM and usual care, or among AYAs with cancer and their age-matched peers without cancer, providing immediately accessible positive psychology skills training can promote longer-term well-being. Second, early response matters. Those who build positive psychological and other coping skills during periods of high stress may also be building mastery over time; they seem to have durable positive outcomes. It follows that those who continue to struggle despite interventions like PRISM might require escalated levels of care. Third, we cannot determine if and how programs like PRISM affect psychological distress. Perhaps they shorten the duration of distress. Perhaps they minimize the severity or duration of distress. Perhaps they do not factor into distress. Additional studies must continue to assess the trajectories of negative affect while also considering the buffering effects of positive psychological skills. Similarly, future work must evaluate strategies to augment and sustain early response, and to intervene among those who start to deteriorate in their well-being over time. Only such comprehensive paradigms will successfully capture holistic approaches to patient well-being.

## Conclusions

The experience of cancer during adolescence and young adulthood is inherently stressful, disruptive to development, and associated with a lifetime of psychosocial risk. It is time we expand our intervention strategy beyond the avoidance of distress and include the protective aspects of positive psychology. In this long-term follow-up evaluation of the PRISM program, we found that those who responded with positive psychological change immediately after the intervention may have experienced durable changes in their overall well-being. Thus, programs like PRISM, which target all 3 domains of AYA well-being during times of high stress, may be a first step toward improving outcomes in this important population.

## Notes

Supplement 1.
Trial Protocol

Supplement 2. **eTable.** Mean Participant-Reported Instrument Scores at Time of Enrollment, 6 Months, 12 Months, and 24 Months

Supplement 3.
Data Sharing Statement
