# Supplementary material for: Assessment of the Promoting Resilience in Stress Management Intervention for Adolescent and Young Adult Survivors of Cancer at 2 Years: Secondary Analysis of a Randomized Clinical Trial
Source: JAMA Netw Open. 2021 Nov 24;4(11):e2136039. doi: 10.1001/jamanetworkopen.2021.36039 (PMC8613597; doi:10.1001/jamanetworkopen.2021.36039)
Supplement: Supplement 1. — Trial Protocol [file jamanetwopen-e2136039-s001.pdf]

Rosenberg AR, Zhou C, Bradford MC, et al. Assessment of the promoting resilience in stress management intervention for adolescent and young adult survivors of cancer at 2 years. *JAMA Netw Open.* 2021;4(11):e2136039. doi:10.1001/jamanetworkopen.2021.36039

Study Protocol

A Pilot Randomized Controlled Trial of the Promoting Resilience in Stress Management (PRISM) Intervention for Adolescents and Young Adults with Cancer

Principal investigator:

Abby R. Rosenberg, MD, MS (Pediatrics, Seattle Children's Hospital)

Co-Investigators:

K. Scott Baker, MD, MS (Pediatrics, Seattle Children's Hospital)

Joyce Yi Frazier, PhD (Clinical and Translational Research, Seattle Children's Hospital)

Research team:

Krysta Barton, PhD

Miranda Bradford, MS

Nicki Sherr, MPH

Kaitlyn Fladeboe, MA

Courtney Junkins, PsyD

Nancy Lau, PhD

Samantha Scott, BA

Angela Steineck, MD

Mallory Taylor, MD

Chuan Zhou, PhD

Study site: Seattle Children's Hospital

Protocol version: 3/6/2019

Contents

List of Abbreviations 3
Research Synopsis 4
Background and Significance 5
Objectives 7
Primary Objective 7
Secondary Objectives 7
Study Design and Methodology 8
Patient Population, Screening and Enrollment 8
Inclusion/Exclusion Criteria 8
Procedures .... **Error! Bookmark not defined.**9
Analyses 13
Timetable and Timelines 16
Informed Consent Process 19
Privacy and Confidentiality 19
Risk/Benefit 20
Risk to participants 20
Benefits to participants 18
Data Safety Monitoring 20
Conflict of Interest 21
Publication and Presentation 21
References 21

APPENDICES (separate attachments):
1. Focus Group Interview Guide
2. Sample "Cheat Sheets" for patients
3. Text for monthly "booster" contacts
4. PRISM Suicide Protocol
5. PRISM Training Manual and Fidelity Scoring
   a. Scripts
   b. Role-playing scenarios for training sessions
   c. Fidelity Tool

List of Abbreviations

AYA: Adolescent and Young Adult

CRA: Clinical Research Associate

PRISM: Promoting Resilience in Stress Management

RCT: Randomized Controlled Trial

Research Synopsis

Study Title: **A Pilot Randomized Controlled Trial of the Promoting Resilience in Stress Management (PRISM) Intervention for Adolescents and Young Adults with Cancer**

Study Population and Sample Size: Adolescent and Young Adult (AYA) patients (ages 12-25 years) with diagnosis of new cancer (between 1 and 10 weeks prior to enrollment) or any history of recurrent or refractory cancer: n=100, stratified by age (12-17 years and 18-25 years)

Study Design: Pilot (phase 2) randomized controlled trial (RCT).

Primary Objective: To test the efficacy of the "Promoting Resilience in Stress Management" (PRISM) among Adolescents and Young Adults with cancer.

Primary Outcome: Change in patient-reported resilience (based on score of standardized Connor-Davidson Resilience Scale) at 6 months.

Secondary Outcomes:

(1) Patient-reported resilience at 12 months
(2) Patient-reported self-efficacy, benefit-finding, psychological distress, quality of life, and health-behaviors at 6 and 12 months.
(3) Qualitative assessment of patient-reported goals at 6 and 12 months
(4) Development of a cohort of AYA cancer survivors for assessment of long-term psychosocial outcomes

Study Duration: 5 years

Background and Significance

The experience of serious illness among adolescents and young adults (AYAs) is unique because they face distinctive developmental challenges, transitions, and choices related to education, employment, identity, relationships and family.[1] Among cancer survivors, AYAs have significantly greater psychological distress and fewer positive health beliefs than younger pediatric or older adult survivors.[2] More than 50% do not pursue adequate cancer-related follow-up care,[3] and ongoing physical, social and emotional challenges such as physical impairment, infertility, uncertainty, fears about recurrence, interruption of life plans, and discrimination in employment and insurance are prevalent.[4,5] In fact, approximately 25% of AYA survivors manifest post-traumatic stress disorder (PTSD), and up to 90% have isolated symptoms of post-traumatic stress.[6]

Although this seems to paint a grim picture for AYAs with cancer, the much less-studied side of the coin shows that positive resources can greatly mitigate these negative outcomes. Age-specific interventions are urgently needed to promote these positive resources and outcomes. Resilience, for example, is a construct describing an individual's capacity to maintain psychological and/or physical well-being in the face of stress, and is a good candidate to buffer the negative effects of stress.[7] While few studies have described positive outcomes in AYA patients with chronic disease, and fewer still have suggested mechanisms to promote resilience,[8] evidence suggests promoting "resilience-resources" (e.g., stress-management and goal setting skills, meaning-making and positive-reframing of negative experiences), as a means to manage stress may be valuable.[9] For example, stress-management interventions are associated with better outcomes, including reduced depression and increased benefit-finding in patients with cancer.[10] Individual differences in goal-seeking and problem-solving have been associated with psychosocial well-being,[10,11] Mindfulness-based interventions have been shown to mitigate emotional distress, fatigue and improve quality of life.[12,13]

Our group has previously described factors of resilience among parents of children with cancer and demonstrated that self-perceptions of resilience (e.g., parent-reported definitions thereof) are strongly associated with outcomes.[14-16] Current studies are examining AYA patient-reported perspectives of resilience resources and the impact of those resources on coping and psychosocial function (SCH IRB-approved study 14067, "Resilience in Adolescents and Young Adults with Cancer.") We have previously piloted and refined the PRISM among AYA patients (ages 12-25) with cancer as well (SCH IRB-approved study 14540).[17,18] Using our prior findings and existing resilience-based theories, we adapted depression and/or stress-prevention interventions that have been successful in other populations[10,11,19-22] to create the Promoting Resilience in Stress Management (PRISM) intervention (Table 1).

Table 1. PRISM intervention session details.

	Topic	Details
**Sessions 1 & 2: Managing Stress**	• Stress management	Mindfulness strategies including: Deep breathing techniques, relaxation strategies, mindfulness meditation, discussion of mindfulness versus mindlessness and over-thinking, acceptance via observing emotions without judgment and acknowledging them
	• Goal setting	Setting specific, realistic, desirable goals, planning for roadblocks, strategies for dealing with roadblocks, identifying how parent/caregiver can help meet goal
**Sessions 3 & 4: Building Resilience**	• Cognitive restructuring	Recognizing negative self-talk, identifying unrealistic/negative thoughts, replacing these thoughts with positive/manageable ones
	• Benefit finding	Reframing current experience into a meaningful one, self-reflection/mindfulness, journaling
**Session 5: Coming Together**	• Reflection	Discussion of strategies practiced from Sessions 1 and 2
	• Celebration	Identification and recognition of successes
	• Resources	Identification of further needs, referrals to additional resources
	• Sharing	Shared conversation with parents: what works, how family can help

This is a patient-focused intervention, directed at the AYA yet inclusive of the family as a whole. The goal in designing the intervention is to offer practical skills to bolster resilience resources in the context of chronic disease management. Thus, the intervention can be adapted to other chronic illnesses. This brief, "in clinic" intervention incorporates concepts of traditional cognitive behavioral therapy (CBT), Acceptance Based Therapy (ACT), and mindfulness-based cognitive therapy (MBCT). Each session can be delivered in conjunction with clinic visits or in-patient stays at our tertiary care center. This approach is uniquely designed to avoid attrition or participation unwillingness often seen in traditional lengthy group CBT and MBCT formats.[23] The present study will provide novel information regarding the intervention's efficacy and inform the development of larger (multi-site) RCTs.

The overall goal of the PRISM intervention is to improve AYA self-perceived resilience, thereby reducing AYA distress, improving quality of life, and minimizing risky health behaviors. The brief format intentionally avoids the extended duration of traditional CBT models of intervention and attempts to maximize patient and family participation. It involves 4, 50-minute, one-on-one sessions approximately 2 weeks apart, plus a 5th parent-inclusive session 2-4 weeks following session #4. These are complimented with age-specific handouts describing the skills and opportunities to practice them, as well as "booster" sessions once monthly. The intervention can be administered by a trained bachelors or masters-level non-clinical professional, as has been effective in similar studies.[24] Specifically, the intervention incorporates four primary skills: 1) stress management/mindfulness; 2) goal-setting; 3) cognitive restructuring; and, 4) meaning-making/benefit finding. Pilot data confirm the PRISM is feasible to conduct, and that patients and parents find it valuable. In addition, preliminary analyses from the first 11 patients enrolled suggests resilience scores (as defined by the 10-item Connor-Davidson Resilience Scale) improve following the intervention (average change in score + 3 points, $p=0.02$).

The rationale for our patient selection reflects both pilot and patient-reported experience in the above mentioned studies, as well as practical, "real-world" AYA oncology clinical needs. In prior studies, AYAs stated that resilience interventions would be most effective at the time of initial diagnosis. Hence, we will offer the study to all newly diagnosed patients (between 1 and 10 weeks from diagnosis). In practical experience, especially within tertiary centers like the Seattle Children's Hospital Cancer and Blood Disorders Center with its dedicated AYA oncology program, many patients are referred with prior histories of refractory or recurrent disease. Discussion with these patients suggests they, too, would be interested in the PRISM studies. Because we are interested in exploring the impact of the intervention during periods of high stress, we will offer the study to all patients who experience the stress of refractory/recurrent disease, regardless of timing. The two age-based cohorts were selected based on known developmental coping styles which vary amongst adolescents compared to younger adults.

Objectives

Primary Objective

To test the efficacy of the PRISM intervention among AYA oncology patients 6 months following enrollment.

Hypotheses: The PRISM intervention will be associated with higher self-perceived resilience, as defined by the 10-item Connor-Davidson Resilience Scale (CD-RISC).

Secondary Objectives

1) ***To explore the efficacy of the PRISM intervention among AYA oncology patients at 12-months following enrollment***

   Hypotheses: The PRISM intervention will be associated with higher self-perceived resilience, as defined by the 10-item CD-RISC.

2) ***To explore the effect of AYA patient-reported self-efficacy, benefit-finding, psychological distress, and quality of life 6- and 12-months following enrollment***

   Expected Findings: Compared to controls, patients who receive the PRISM will have higher reported self-efficacy, benefit findings, and quality of life scores. Similarly, they will have lower psychological distress and endorse fewer risky health behaviors.

3) ***To qualitatively explore AYA patient-reported goals 6- and 12-months following enrollment.***

   Expected Findings: Compared to controls, patients who receive the PRISM intervention will describe more concrete and achievable goals.

4) ***To develop a cohort of AYA cancer patients (to be followed longitudinally) for interval assessments of psychosocial outcomes and health behaviors following cancer therapy.***

Study Design and Methodology

We will conduct a pilot randomized controlled trial (RCT) among 2 cohorts of AYA patients with newly diagnosed or recurrent cancer: (1) ages 12-17 years; and (2) ages 18-25 years (total N=100).

Eligible patients will be 12-25 years old, either between 1 and 10 weeks from their diagnosis of new cancer OR with any history of refractory/recurrent disease, and receive their cancer-care at SCH. SCH treats approximately 75 new AYA cancer patients each year. Approximately 80% of our AYA patients enroll on clinical trials, including survey- and interview-based studies.

Patient Population, Screening and Enrollment: Over the first three years of the study, approximately 150 newly diagnosed AYA cancer patients will be eligible for each cohort of the study. We will identify eligible patients through existing screening methods used in the hematology/oncology clinic. Specifically, trained and dedicated research associates will screen clinic patients for eligibility. We will request a waiver of consent for screening purposes for this study, such that CRAs may identify potential and eligible patients prior to their scheduled clinic visits. We will then contact patients' primary providers for all screened-eligible patients to verify appropriateness and to introduce the study. Study personnel may then contact patients and their parents to discuss the project, and answer questions by phone (and in advance of their clinic visit) or while at a regularly scheduled outpatient visit or inpatient. Should families prefer to continue to discuss the study in person, we will arrange a follow-up conversation regarding the study at the time of their clinic visit. This is a system that has been in place and worked successfully for over a decade at SCH. All patients over 18 will provide signed informed consent prior to enrollment. Those between the ages of 12 and 17 will provide signed assent and must also have consent from their designated parent or guardian. Our enrollment target will be 100 total patients with recently diagnosed or recurrent disease (estimated ~50 in each age-cohort).

If patient is no longer coming to clinic on a regular basis and turns 18 years old or needs to re-consent due to a modification with the protocol (or other cause), study staff will mail the consent to the participant and will call the participant (and parents if applicable) and go over study changes. The consent mailing will include a pre-paid return mailing envelope so the participant can sign and return to return to study staff.

Patients who speak English but have non-English speaking parents will be eligible to participate. Procedures for screening, approach, and enrollment of these families will be similar to above, except as follows:

For Spanish Speaking Parents:

(1) **For patients < 18 years-old**: Spanish-English translations of the consent and assent forms will be provided for parents during discussions of the study and consent conferences and all discussions will occur with trained medical interpreters.

(2) **For patients 18 years and older**: Spanish-English translations of the consent will be provided for parents during discussions of the study and consent conferences at patients' requests. Likewise, if patients wish their parents to participate in session 5, "coming together," parents will be invited to sign a Spanish version of the consent. All discussions involving Spanish (non-English) speaking parents will occur with trained medical interpreters.

(3) **For all patients**: Session 5 ("coming together") will be offered in the presence of a Spanish interpreter. All other 1:1 sessions will still be conducted in English with English-speaking, trained interventionists. (Note future studies will explore iterations of the full intervention in Spanish).

For Other Non-English Speaking Parents, on a case-by-case basis:

(1) Discussions of study will occur with interpreters in the appropriate language. If families and patients are interested in participation, informed consent forms would be translated into the native language of the parent.

(2) Everything pertaining to Spanish speaking parents would be the same for other non-English speaking parents except that discussions, conferences, and Session 5 would be in the native language of the parent.

Inclusion/Exclusion Criteria

Inclusion Criteria:

1) **Age 12-25 years**
   a. **Patient aged 12-17 years: has signed informed assent and their parent/legal guardian has signed informed consent for study participation.**
   b. **Patient aged 18-25 years: has signed informed consent for study participation.**
2) **Diagnosis of malignancy treated with chemotherapy and/or radiation therapy at SCH**
   a. **New diagnosis of malignancy within 1-10 weeks of enrollment**
   b. **New diagnosis of recurrent disease (after initial remission) or refractory disease at any time during therapy**
3) **Patient able to speak and read English language**
4) **Cognitively able to participate in interactive interviews**

Exclusion Criteria:

1) **Patient refusal to participate (any age), or parental refusal to participate for patients less than 18 years of age**
2) **Cognitively or physically unable to participate in interactive interview**
3) **Patient unable to speak and read English language**
4) **Patient without chemotherapy and/or radiation therapy as part of cancer treatment (e.g., surgical resection only patients are not-eligible).**

Procedures

1) RANDOMIZATION: Upon Enrollment, patients in each cohort will be randomized in a 1:1 ratio to receive the PRISM at study entry ("experimental" arm). Standard non-directed supportive care will be provided for all patients, including an assigned social worker throughout cancer treatment. Each Cohort will have its own randomization: 1) patients ages 12-17; and 2) patients ages 18-25. Participants will be randomized in a 1:1 ratio to control or PRISM in each cohort. Throughout the screening period until allocation of the control or PRISM, participants will be assigned a screening number, according to the chronological order of screening. The screening number will be used as the participant ID throughout the study.

   The randomization algorithm will be constructed by the study statistician using a permuted blocks scheme with varying block sizes; only the statistician will be aware of the block sizes until completion of the study. Separate randomization schedules will be used for the two cohorts and all randomizations will be stratified by new/recurrent disease. We will not stratify by specific diseases or by disease status (new or recurrent disease), but will collect this variable and adjust analyses accordingly (see below). A statistician independent of the study will prepare the final randomization list, which will be administered by a clinical research associate independent of the study using REDCap. As a quality control measure, a randomization log will be maintained by a separate CRA to track the participant ID, stratum, randomized assignment, and date of randomization.

2) The RESILIENCE IN PEDIATRIC CANCER ASSESSMENT (RPCA): Enrolled patients will complete the Resilience in Pediatric Cancer Assessment Instrument (RPCA) within 4 weeks of enrollment, and then 6-, 12-, 24-, 36-, 48-, and 60- months later (Table 2). An abbreviated version of the survey, containing only the Connor-Davidson Resilience Scale (CD-RISC) will be used at the 6- and 12-week time-points. The complete RPCA has been previously developed and refined in prior studies at SCH ("Understanding Resilience in Parents of Children with Cancer," IRB 13551) and "Resilience in Adolescents and Young Adults with Cancer," IRB 14067). It includes a total of 131 items and takes approximately 20-25 minutes to complete. At the 36-, 48- and 60-month RPCA, there will be an additional 91 questions added, resulting in 222 items. The RPCA includes validated instruments to assess patient resilience, self-efficacy and goal-setting skills, benefit-finding, psychological distress, quality of life, risky behaviors and demographic variables:

**Table 2. The Resilience in Pediatric Cancer Assessment (RPCA)**	
Resilience (CD-RISC)<br>Self-Efficacy & Goal-Setting<br>- HOPE<br>- Qualitative Goals<br>Benefit-Finding (BFSC)	Psychological Distress<br>- K6<br>- HADS<br>Quality of Life (PedsQL)<br>Risky Behavior (GAPS)<br>Demographics
CD-RISC: Connor-Davidson 10 Resilience Scale[25]; HOPE: Synder Hope Scale[26]; BFSC: Benefit Finding Scale for Cancer[27]; K6: Kessler-6 Psychological Distress Scale[28]; HADS: Hospital Anxiety and Depression Scale[29]; PEDS-QL: Pediatric Quality of Life Cancer Module Teen Report[30]; GAPS: Guidelines for Adolescent Preventative Services adapted risk assessment[31]; IOC: Impact of Cancer scale; HCSE: assessment of Healthcare Self-Efficacy.	

a. CONNOR-DAVIDSON RESILIENCE SCALE (CD-RISC-10)
The Connor-Davidson Resilience Scale is a reliable and widely used instrument to measure inherent resiliency.[25] Questions revolve around personal problem-solving and approaches to adversity. The 10-item instrument has high internal consistency (Cronbach's alpha = 0.85), and has been used in diverse populations including adolescents, parents and cancer patients.[25,32] Correlative studies have evaluated the scale with other psychosocial measures such as psychological distress,[33] PTSD,[34] and social support.[35] It also has been used in pharmacologic and other intervention studies to model modifiable

outcomes Each item consists of a 5-point Likert scale (scored from zero to four) for total of 40 points. The mean score among well US adults is 31.8, with higher scores reflecting greater resilience.

b. HOPE SCALE
The Hope Scale contains 8 hope items plus 4 "filler" questions, and measures "the overall perception that one's goals can be met."[26] The instrument distinguishes between the ability to generate a route to one's goals (termed "pathway" thoughts) and the ability to initiate and maintain the actions necessary to reach a goal (termed "agency" thoughts). Prior studies performed among AYA cancer patients have shown that high-hope individuals have improved psychosocial outcomes.[36] The instrument has been validated in both adult and pediatric settings and is scored on an 8-point Likert scale. Higher scores imply greater levels of hopeful thought patterns. The mean scores among well college students is 25 (SD 3.0). Cronbach's alphas for the whole scale range from .74 to 0.84. In addition, the scale has demonstrated good test-retest reliability over time (correlations at 3-, 8-, and 10-week intervals are 0.85, 0.73, and 0.76, respectively (all $p<0.001$).

c. QUALITATIVE GOALS
In order to assess learned skills of concrete goal-setting (in addition to perceived self-efficacy which is measured with the HOPE scale, above), the RPCA includes 3 open-ended questions regarding patients' goals for the next several months to years. For (a) the next few months, and (b) the next few years, each patient is asked to give an example a goal he/she hopes to accomplish, as well as how he/she expects to accomplish it. Qualitative responses will be de-identified and scored by 2 independent reviewers who are blinded to the patients' randomization arm, as follows. First, reviewers will assign 1 point each for the goals that are (i) concrete; (ii) actionable; (iii) described with steps/pathways to completion; (iv) described with possible pitfalls and alternatives. (All of these items are taught in the PRISM goal-setting session). Inter-rater reliability will be tracked. When two reviewers agree, the numeric score (range 0 to 4)will be added to the database. If reviewers disagree, a third blinded reviewer will provide an additional score and the average of all three will be entered into the database. Second, reviewers will analyze all goals with open-ended coding and grounded-theory techniques[37] to ensure appropriate interpretation and translation of findings. Please see statistical analysis section for details.

d. BENEFIT FINDING SCALE FOR CHILDREN
The Benefit Finding Scale for Children[38] was adapted by pediatric psychosocial clinicians from the Benefit Findings Scales used for adult patients with cancer.[27,39,40] Specifically, items were assessed for their relevance to pediatric, adolescent and young adult cancer patients and pediatric psychologists provided consensus opinion regarding relevance and appropriateness for this population. Several items were further adapted with minor re-wording, while others were created to capture comparable issues. Ten of the items of the final scale depicts a potential benefit of illness, and 10 depict potential burdens. All are answered on a 5-point Likert scale ranging from "not at all" to "very much." The internal reliability (Chronbach's alpha) of the scale is 0.83. Reported total mean score among pediatric and AYA cancer patients is 37.35 (SD 7.8, range 12-50).

e. KESSLER-6 GENERAL PSYCHOLOGICAL DISTRESS SCALE (K6)
This 6-item scale measures "level of psychological distress experienced in the past month." It was developed for the US National Health Interview Survey,[28] and is currently being used in Canada, Australia and world-wide as part of the World Health Organization (WHO) world mental health initiative.[41] The instrument strongly discriminates between community cases and non-cases of Diagnostic and Statistical Manual of Mental Disorder (DSM)-IV psychiatric disorders such as serious

emotional distress or serious mental illness (area under the curve [AUC] = 0.74-0.88).[42] It has been extensively cross-validated, including among adolescents.[43] Responses are scored on 5-point Likert scale, generating a range of zero to 24 points. Previous studies have shown that scores ≥ 7 are consistent with "high" distress and those ≥ 13 meet criteria for serious, or debilitating psychological distress.[44] The mean score among well US adults is approximately 3 and the prevalence of serious distress is 2-4%; however, our recent studies have shown that mean scores among parents of children with cancer may be significantly higher.[45]

f. HOSPITAL ANXIETY AND DEPRESSION SCALE (HADS)
The Hospital Anxiety and Depression Scale (HADS) was developed to assess anxiety and depression in patients with serious illness.[29] It deliberately avoids reliance on aspects of these disorders which are also common somatic symptoms of illness (e.g., fatigue, insomnia). It has been validated in young adults with chronic illness[46] as well as pediatric and adolescent cancer survivors.[47] The scale has excellent reliability (alpha for anxiety and depression subscales 0.32 and 0.82, respectively). It consists of 7 questions for anxiety and 7 for depression. Each item is scored from 0-3, for a total range of 0-21 points per subscale. Caseness of anxiety and depression are each defined as 8 of 21 points, with sensitivity/specificity of 0.8/0.9 for anxiety and 0.8/0.8 for depression.[29]

g. PEDIATRIC QUALITY OF LIFE (PEDS-QL) GENERIC & CANCER MODULE TEEN REPORTS
The PedsQL 4.0 Generic and the PedsQL 3.0 Cancer Module Teen Reports include a total of 42 items evaluating health-related quality of life specific to adolescents with cancer. The generic module asks about general health-related activities, emotional, social, and school well-being. The cancer module also assesses: pain and hurt; nausea; procedural anxiety; treatment anxiety; worry; cognitive problems; perceived physical appearance; and communication. There are scales available for teens and young adults.[30,48] The Teen Reports have been used successfully in prior research investigating HRQOL in adolescents and young adults with cancer with low rates of refusal and minimal missing data,[49] and the young adult module has recently been added successfully to cooperative group studies within the Children's Oncology Group (personal communication with Lilian Sung, chair of Cancer Control Committee, 11/2013). Individual items for the Generic Core Scales and Cancer Module are rated on a 5-point Likert scale and total scores are transformed to a 0-100 scale with higher scores representing better quality of life. The total scale score represents a mean of the individual item scores. At least 50% of the total items must be completed to compute a total score. Total scores for the individual scales are computed by taking an average of the number of items completed within the scale.[30] Previous research suggests that the Generic Core Scales and Cancer Module can be easily completed by AYAs undergoing treatment for cancer.[30,49] Internal consistency of the total score of the adolescent Generic Core Scales is reported as 0.92 in a combined sample of healthy adolescents and adolescents with cancer. Internal consistency of the individual subscales ranged from 0.75 to 0.88.[30]

h. AMERICAN MEDICAL ASSOCIATION GUIDELINES FOR ADOLESCENT PREVENTATIVE SERVICES (GAPS)
The GAPS survey includes not only validated questions appropriate to assess risky behavior and needs of adolescents and young adults, but also individual recommendations for specific supportive care measures and interventions based on patient-response.[31] We have adapted 12 items to query patient lifestyle as well as needs for multidisciplinary services such as reproductive health, gynecology, substance abuse counseling, psychiatric counseling and other supportive services.

i. IMPACT OF CANCER (IOC) SCALE

The 91-item Impact of Cancer scale for AYAs measures both positive and negative perceptions of the impact of cancer experiences on AYA patient-centered outcomes including social life, uncertainties, worries and wonders, cognitive function, sense of purpose/life goals, identity, health behaviors and health literacy. Conditional scales for relationship concerns are triggered based on respondents' relationship status. Items are assessed with a 5-point Likert Scale. Means are calculated for each subscale and two overarching positive and negative impact scores are calculated with higher scores indicative of a greater (positive or negative) impact on each.[50,51]

j. HEALTHCARE SELF EFFICACY ASSESSMENT

The 3 question HCSE scale includes three items related to survivors' perceived confidence in discussing concerns with their physicians, making appointments with physicians, and obtaining cancer follow-up care in the next 2 years. Responses are comprised of a three-point Likert-type scale ranging from "not confident" to "very confident". Items are summed to form a continuous composite score (ranging from 3 to 9), with higher scores indicating greater HCSE.[52]

k. DEMOGRAPHICS

Demographic data will be self-reported and will include age, gender, race/ethnicity, level of education and, for parents, household income. Economic survey questions were adapted from the US Household Food Security Survey Module,[53,54] the Children's Health Watch Survey,[55] and the SCCC.

To ensure data quality, a research associate (RA) will review RPCA surveys within 48 hours of their completion for missing fields and will call participants to clarify as warranted. All surveys will be available for completion in person, by paper, or online via the REDCap system, a secure HIPAA-compliant, high-quality data collection tool. We will schedule each participant for dedicated survey-completion appointment in tandem with other scheduled oncology visits. We will meet patients in person or send them an email invitation to complete their survey via REDcap. Those who fail to complete their surveys within 1 week will receive a phone call from the research associate. They will receive a $25 incentive following completion of the baseline survey, a $50 incentive following completion of the 6-month survey, and a $50 incentive following completion of the 12-month survey. The higher incentives at the follow-up points are to promote maximal response rate at the primary (6-month) and secondary (12-month) endpoints, but also to reduce patient attrition over time. An additional $25 will be provided following each yearly RPCA survey completion, for up to an additional $100 over the course of 4 years.

Surveys may be completed in person or by phone (verbally), online using RedCap, or by paper-and-pencil. Participants will be offered all options. Participants will be given the survey prior to their first PRISM session and asked to complete it before the session begins. The abbreviated (aRCPA; CD-RISC instrument only) surveys will be administered at 6-8 and 10-14 weeks; follow-up surveys will be administered at 6-months,12-months, 24-months, 36-months, 48-months, and 60-months following enrollment for both cohorts. If by chance the participant is on the experimental arm and has not completed all 5 PRISM sessions by 6 months, we will still request the survey to be competed at 6 months, prior to completion of the study visits.

Table 3. Timeline of evaluations for PRISM intervention and standard of care arms

*Time from Enrollment**	*0-4 weeks*	*4-6 weeks*	*6-8 weeks*	*8-10 weeks*	*10-14 weeks*	*Up to 6 months*	*12-60 months*
**Session Schedule** (Experimental Arm)	PRISM 1	PRISM 2	PRISM 3	PRISM 4	PRISM 5**	Monthly "boosters"	

Time from Enrollment	*0-4 weeks*		*2 months*		*4 months*		*6 months*	*12 Months*	*12-60 months*
**Survey Schedule** (Control and Experimental arms)	RPCA		aRPCA		aRPCA		RPCA	RPCA	Yearly RPCA

RPCA: Resilience in Pediatric Cancer Assessment;
aRPCA: abbreviated RPCA;
PRISM: Promoting Resilience in Stress Management Intervention
* Dates are approximate and are dependent on the actual date of the previous PRISM session.
**Optional

3) The PROMOTING RESILIENCE IN STRESS MANAGEMENT (PRISM) INTERVENTION: Enrolled patients randomized to the experimental arm will be invited to schedule and complete the PRISM intervention. The total intervention consists of four, 30-50 minute 1-on-1 sessions approximately 2 weeks apart plus a fifth session for patients and their caregivers, together (Tables 1 & 3). The intervention is administered by a trained, bachelors or masters-level non-clinical professional as modeled from previous models.[56-58] In our pilot study, we have successfully trained three interventionists who have all mastered delivery of the intervention with high fidelity. A chronic illness social worker or psychologist will train all new participating staff in the intervention, with clinical oversight from the study PI. (Please see training manual in the appendix.) The first session will occur in person, and within 4 weeks of their enrollment. Due to the variable medical conditions of this population, we expect that there may be medical complications that may prevent enrolled participants from completing their first session within 4 weeks of enrollment. The PI and on-service attending physician will review medical status on a case by case basis and if deemed medically inappropriate to continue at that time, participation will be placed on hold and resumed when complications are resolved. The second through 5th sessions will be scheduled around patient clinic and/or hospital visits and will be conducted in person wherever possible. At patient-specific requests, these latter sessions may be conducted by Skype, or phone. This will be done to maximize the number of families who will be able to participate. We do not want returning to the clinic to be a prohibitive factor from participating. (Consults via Skype have previously been approved by the Institutional Review Board at Seattle Children's Hospital.)

Details of the sessions are listed in Table 1 (page 5). Briefly, session 1 ("Stress-management") focuses on mindfulness skills including deep breathing and relaxation techniques, and building awareness and acceptance of stressors. Session 2 ("Goal setting") teaches simple goal-setting skills (e.g., identifying realistic, concrete and actionable goals, planning steps towards their achievement, preparing for roadblocks and identifying alternative pathways). Session 3 ("Cognitive Restructuring") trains patients to recognize negative emotions and demoralizing self-talk and helps them develop skills to reframe these in a positive light. Session 4 ("Benefit Finding") focuses on finding meaning and/or benefit from difficult situations (including cancer). Finally, the optional session 5 ("Coming together") allows patients

to reflect on the skills they have learned, to identify those that resonate and work for them, and to share their thoughts with parents. During this last session, parents are explicitly asked to join and listen to the discussion. Study personnel will review and share with parents the explicit skills endorsed by patients and encourage shared conversation about how parents/patients can support one another. If the patient does not wish to have a "coming together", a booster session will take the place of session 5. As above, for families where parents prefer Spanish or another non-English language, this 5th session will be conducted with a trained interpreter of the native language of the parent..

All of the sessions of the PRISM will be audio-recorded as possible, barring issues with the recorder or refusal of the patient to be recorded. The answers of the 4 feedback questions at the beginning of the 4 consecutive sessions will be transcribed and saved without identifying data for research purposes. Administrators of the PRISM will explain that the sessions will be taped and reviewed by the study team with the goal of assessing adherence to the protocol, inclusion of required elements, and presence/absence of additional information with the exception of the feedback questions. As possible, the PI or a study CRA will review the first 5 sessions for each interventionist, and score them for fidelity using a standardized tool (see appendix.). After the first 5 sessions are reviewed for each interventionist, fidelity checks will occur for each interventionist regularly and as-needed. Intervenors will receive feedback regarding adherence to protocol and approach will be refined if needed.

Participants on the intervention arm will also receive once monthly "booster" contacts until they reach the 6 month point from enrollment. These will include brief (10-20 minute) in-person contacts in clinic, in the hospital, by phone, or by email and will consist of opportunities to practice specific skills (at the patient's discretion). Study staff will contact patients to coordinate such visits and will prompt them by asking: "Would you like to review or practice any of the resilience skills?" [If needed, staff will remind patients of all 4 sessions. If patients are willing, study staff will ask,] "Which one?"

In order to optimize engagement, patients in both arms of the study will be asked to complete the abbreviated RPCA at 6-8 weeks and 10-14 weeks post-enrollment (prior to sessions 3 and 5 for participants on the PRISM arm). For those on the non-intervention arm of the study, staff will schedule a "study check-in" visit to coordinate the aRPCA completion and thank families for their continued participation.

In both arms of the study, if any participant declines to complete the 6-month RPCA survey, the abbreviated version of the questionnaire will be offered in an effort to collect full data for primary endpoint assessment.

Participants in both arms of the study will be contacted on a yearly basis for up to four years after their first year of participation. At that time, participants will be asked to complete RPCA surveys. Participants will be contacted by phone, email or mail annually to receive their RPCA survey. If we have not received a response within a week of initial contact, participants will receive a follow-up phone call.

At each respective study time point (PRISM session and survey completion), study staff will complete a brief medical record review of each participating patient to assess current health status. Data for the chart review may be supplemented by data-requests from Knowledge Management, as needed.

4) ANALYSES

Our target sample size for this pilot RCT (total N=100) was based on conservative estimates of completing patient accrual over three years. During that time-frame, approximately 150 new patients will start their therapy at SCH. Based on prior experience of 75% enrollment rates, and accounting for

an additional 10% attrition, we expect approximately 100 potential patients with new diagnoses to be eligible and to complete the study. During the same time-frame, we anticipate approximately 40 patients to present with newly recurrent disease. Again, assuming 75% enrollment and 10% attrition, we would expect 27 patients to be eligible, enroll, and complete the study.

Power calculations adjusted for approximately 10% attrition in each study arm and confirmed the above-described target sample size was also sufficient to detect meaningful differences in the primary outcome between groups. Preliminary data from our prior studies (the Resilience in Adolescents and Young Adults [RAYA] study), indicate that CDRISC scores are normally distributed with mean scores among newly diagnosed AYAs = 31 (SD=5.3, based on n=23). In the entire cohort (n=45 experimental subjects and n=45 controls), we will be able to detect a true difference in the experimental and control subjects of +/- 3.2 points with probability (power) 0.8 and +/- 3.5 points with probability (power) 0.9. Among each stratum (n=22 experimental and n=22 controls), we will be able to detect true mean differences of +/- 4.6, and +/- 5 points with power of 0.8 and 0.9, respectively. The Type I error probability associated with these tests of the null hypothesis that the group means of the experimental and control groups are equal is 0.05. Additional power calculations for selected secondary outcomes are shown in Table 4.

Table 4. Power calculations for selected outcome measures.

**Sample Size***	**Power**	**CD-RISC** (mean 31, SD 5.3)	**BFSC** (mean 37.4, SD 7.8)	**K6** (mean 5.4, SD 3.0)	**PEDSQL** (mean 55.4, SD 11.7)
45/45	80%	+/-3.2	+/-4.7	+/-1.8	+/-7
	90%	+/-3.5	+/-5.4	+/-2	+/-8
22/22	80%	+/-4.6	+/-6.7	+/-2.6	+/-10
	90%	+/-5	+/-7.8	+/-3	+/-11.7

*experimental arm/control arm
CDRISC: Connor-Davidson Resilience Scale, 10-item score (primary outcome); BFSC: Benefit Finding Scale for Children (secondary outcome); K6: Kessler-6 Psychological distress scale (secondary outcome); PEDSQL: Pediatric Quality of life (secondary outcome).

Statistical analyses will be performed with the Stata 12 software package. A detailed statistical analysis plan will be drafted by the study statistician and PI prior to final analyses. Demographic and clinical characteristics will be summarized overall. Additional items within the RPCA will be summarized at each time point using descriptive statistics appropriate for each item: frequencies and proportions for categorical variables, means and standard deviations for continuous variables, or median and interquartile range if the distribution of the variable is markedly skewed. We will use an “intent to treat” analysis plan to avoid confounding by non-random participant attrition. However, secondary analyses will include examination of effect by intervention completion and will therefore assess differences in CDRISC and other instrument scores based on PRISM completion. Hierarchical linear models[59] will be used to account for the multiple observations taken over time. Linear CD-RISC (resilience) score will be the outcome, and the PRISM intervention the predictor, of interest. A contrast will be constructed, which is a generalization of the Cochran-Mantel-Haenszel test,[60] to examine if there is an increase in CD-RISC scores from baseline for the intervention group compared to the control group at the primary time point of interest, 6 months. Additional contrasts will be used to examine group differences at 6-weels, 10-weeks, and 12 months as well as group trajectories of resilience scores over time; similar analyses will examine outcomes of self-efficacy, goal-setting, benefit-finding, quality of life, psychological distress, and risky health behaviors at 6- and 12-months, modeled separately.

Patient-reported goals will be analyzed qualitatively. First, all written responses will be de-identified and transcribed verbatim. Two independent reviewers, blinded to patient's randomization, will code each transcript in two ways: First, a priori scoring will include one point each for goals that are (i) concrete; (ii) actionable; (iii) described with steps/pathways to completion; (iv) described with possible pitfalls and alternatives. When two reviewers agree, the numeric score (range 0 to 4) will be added to the database. If reviewers disagree, a third blinded reviewer will provide an additional score and the average of all three will be entered into the database. Second, following completion of all study collection, reviewers will analyze all goals with open-ended coding and grounded-theory techniques[37] to ensure appropriate interpretation and translation of findings. Pooled themes from patients who received the intervention will be compared with those from controls.

Future Plans:

Findings from this pilot randomized controlled trial will inform the development of larger (multi-site) clinical studies aimed to formally test the intervention, as well as direct future pilot studies (e.g., Spanish version of the intervention). Likewise, findings from the present study may be translated to other settings, impacting more broadly on the care of patients and families in other medical or stressful situations. This study will enable better family-centered care after serious illness and ultimately enable the positive development and well-being of AYA patients and their families.

Timetable for development of work:

Academic Years 2014-2017												
	Q1	Q2	Q3	Q4	Q1	Q2	Q3	Q4	Q1	Q2	Q3	Q4
Human Subjects Approval, Study set-up, training, and initial Recruitment	■	■										
Enrollment			■	■	■	■	■	■				
Intervention sessions/follow-up (3 months total)				■	■	■	■	■	■			
Intervention re-design and fine-tuning based on qualitative feedback								■	■	■		
Manuscript development and dissemination of findings							■	■	■	■	■	■
Follow-up multi-site study design, funding applications							■	■	■	■	■	■

Informed Consent Process

A waiver of consent will be requested for screening purposes only. All patients who screen eligible will provide signed informed consent/assent prior to enrollment.

The consent meeting between the CRA and eligible participants (with parents if applicable) will include an explanation of the study in developmentally appropriate lay-language. For patients/families who are screened and provide verbal interest by phone, the written consent form may be mailed for receipt and review prior to enrollment. English speaking AYA patients with Spanish-speaking parents will be provided both English and Spanish versions of the consent, and all discussions for patients < 18 years of age who have Spanish speaking parents will take place with a trained medical interpreter. All participants and parents will be provided an opportunity to read the consent/assent form in their preferred language (English or Spanish), to ask questions about the study and have those questions answered by the research team member before deciding about study participation and signing the consent/assent form. Parental permission for study participation will be obtained first, then patient assent will be obtained for all patients 12-17 years of age. If a patient indicates that they do not want to participate in the study, that non-assent will override the parent's permission and the patient will be recorded as a refusal. The research team member will redirect any parent who attempts to convince their child to participate in the study and remind them of their child's right as a potential research participant to refuse participation without coercion. Consent to study participation will be obtained from patients 18 years of age and above. The CRA will emphasize to all patients and parents in developmentally appropriate lay-language that being in the study is their choice, that they may choose not to participate or may change their mind at any time and it will not affect how their nurses or doctors care for them.

After signing informed consent/assent, participants will then be randomized and asked to complete the baseline RPCA survey. Those who are randomized to the intervention arm will also be invited to schedule their first PRISM session. As above, for those expressing interest prior to clinic visits, the PRISM session 1 will be scheduled to coincide with the same day of enrollment. However, should patients or families change their minds and decline the study, the scheduled PRISM session will be cancelled at no cost to the family.

This study includes children. Pediatric patients with serious illness are at risk for poor outcomes and may benefit from resilience-enhancing interventions in the future. We justify the inclusion of children in this project because the implementation of those interventions requires feasibility information and patient feedback. This study will provide those crucial data. Patients enrolling in this study may, in fact, benefit from the intervention; however, at the time of consent, we will ensure that all patients and families understand the objective of this study are to test the feasibility of this intervention such that it may be used prospectively in the future (see risks/benefits below).

Privacy and Confidentiality

All information collected for research purposes will be de-identified. Identifying information (names, addresses and phone numbers) will be used initially to identify potential patients for approach. Research records will be stored in locked cabinets and secure computer files. Patients names will be kept on a password protected database and will be linked only with a study identification number for this research. There are no patient identifiers. Feasibility data will represent only frequencies and percents. All data will be entered into a computer that is password protected. Data will be stored in a locked office of the investigators and maintained for a minimum of three years after the completion of the study.

Risk/Benefit

Potential Risks of Participation: The intervention ("Promoting Resilience in Stress Management", PRISM) may address sensitive matters in that it asks patients to identify stressors and negative thoughts. Adolescent and young adult participants may be prompted to think about the threat to their life posed by their cancer, as well as other difficult topics such as suicidal ideations, sexuality and substance abuse. The topics to be covered may provoke sadness, anxiety, depression, fear or doubt. Administrators of the intervention will be trained to recognize and query thoughts of self-harm or harm to others. Should these concerns arise, patients will be referred immediately to their primary social work and medical teams. As part of their informed consent process, patients will be made aware of this policy, as well as the fact that confidentiality may be broken in the case that providers see an immediate threat to the patient's or another's safety. No physical risks are expected to arise from the study.

Procedures to Minimize Risk: Subjects will be informed that they may refuse to answer any questions if they wish and may choose to stop participating at any time. The intervention has been adapted from similar tools used among cancer and diabetes patients, as well as other AYA populations. No previous adverse events have been reported form previous investigations. If an event were to occur in this study, we would try to determine if there were a link between the questions and the adverse reaction and determine if any modification of the survey instrument would be advised. As above, subjects' responses will be monitored carefully and prompt referrals made to the appropriate mental health professional if warranted.

Benefits: We hypothesize that patients who receive the intervention will have diminished psychological distress and greater quality of life. We also hypothesize that parents will benefit similarly from their child's participation because prior experience in pediatric cancer studies suggests it is personally important for some patients and caregivers to share their perspectives, challenges and growth experienced during their cancer. However, there may be no direct benefit for participating in this study if our hypotheses are wrong. More broadly, information gained from this study may heighten the understanding of the AYA cancer experience and elucidate strategies that foster resilience and promote better quality of life in this group of high risk patients. These strategies could be extended to the care of other AYA patients facing non-cancer-related life-threatening illness. This research has the potential to contribute to the research base concerning the promotion of optimal quality of life and mental wellness for all AYA patients.

Alternatives: Patients may opt not to participate in the research. Their care will not be affected in any way should they decline participation.

Data Safety Monitoring

As above, a research associate will report immediate threats to patients' or others' safety to the PI within 24 hours of awareness. In cases of concern for patient or others' safety, immediate referrals will be made to the PI, and the patients' primary medical and social work teams for in-person evaluation or referral to the appropriate mental health professional if warranted. After hours, the PI and on-call providers from the medical teams will be notified. All concerns for patient or other person's safety will be reported as an adverse event (AE) to the IRB within 1 week. In addition, the PI will review the potential risks and reported findings at annual renewals. While this is not a therapeutic trial and we do not anticipated medical complications, we will nevertheless notify the IRB of all participant deaths. The study will be suspended for review if 2 patients or parents report threat to themselves or others.

Conflict of Interest

None of the investigators has any conflict of interest.

Publication and Presentation

Study results will be published in peer-reviewed journals and/or presented at professional meetings.

Data and/or Sample Sharing

Data will not be shared outside the group of investigators conducting the study but will be fully shared during and after the study with investigators in the group. De-identified study data will be banked indefinitely for future use by the group of investigators conducting the study and access will be controlled by the PI's. Future studies will formally test the intervention once its feasibility is confirmed. Should the intervention be effective, it will be made publically available for use by the broader medical communities caring for AYAs with serious illness.

Funding Source

Grant number: KL2 TR002317
UW eGC1 ID: A113694
P.I.: John Amory

References

1. Eiser C, Penn A, Katz E, Barr R. Psychosocial issues and quality of life. *Semin Oncol.* 2009;36(3):275-280.
2. Kazak AE, Derosa BW, Schwartz LA, et al. Psychological outcomes and health beliefs in adolescent and young adult survivors of childhood cancer and controls. *J Clin Oncol.* 2010;28(12):2002-2007.
3. Nathan PC, Greenberg ML, Ness KK, et al. Medical care in long-term survivors of childhood cancer: a report from the childhood cancer survivor study. *J Clin Oncol.* 2008;26(27):4401-4409.
4. Pendley JS, Dahlquist LM, Dreyer Z. Body image and psychosocial adjustment in adolescent cancer survivors. *J Pediatr Psychol.* 1997;22(1):29-43.
5. Richardson RC, Nelson MB, Meeske K. Young adult survivors of childhood cancer: attending to emerging medical and psychosocial needs. *J Pediatr Oncol Nurs.* 1999;16(3):136-144.
6. Kazak AE, Alderfer M, Rourke MT, Simms S, Streisand R, Grossman JR. Posttraumatic stress disorder (PTSD) and posttraumatic stress symptoms (PTSS) in families of adolescent childhood cancer survivors. *J Pediatr Psychol.* 2004;29(3):211-219.
7. Southwick SM, Charney DS. The science of resilience: implications for the prevention and treatment of depression. *Science.* 2012;338:79-82.
8. Haase JE. The adolescent resilience model as a guide to interventions. *J Pediatr Oncol Nurs.* 2004;21(5):289-299; discussion 300-284.
9. Gillham JE, Reivich KJ, Freres DR, et al. School-based prevention of depressive symptoms: A randomized controlled study of the effectiveness and specificity of the Penn Resiliency Program. *J of Consult Clin Psych.* 2007;75:9-19.
10. Carver CS. Enhancing adaptation during treatment and the role of individual differences. *Cancer.* 2005;104(11 Suppl):2602-2607.
11. McCarty CA, Violette HD, McCauley E. Feasibility of the positive thoughts and actions prevention program for middle schoolers at risk for depression. *Depress Res Treat.* 2011;2011:241386.
12. van der Lee ML, Garssen B. Mindfulness-based cognitive therapy reduces chronic cancer-related fatigue: a treatment study. *Psychooncology.* 2012;21:264-272.
13. Van Son JNI, Pop VJ, Blonk MC, et al. The Effects of a Mindfulness-Based Intervention on Emotional Distress, Quality-of-Life, and HbA1c in Outpatients with Diabetes (Diamind): a randomized controlled trial. *Diabetes Care.* 2012;epub ahead of print.
14. Rosenberg AR, Wolfe J, Bradford MC, et al. Resilience and psychosocial outcomes in parents of children with cancer. *Pediatr Blood Cancer.* 2013.
15. Rosenberg AR, Baker KS, Syrjala KL, Back AL, Wolfe J. Promoting resilience among parents and caregivers of children with cancer. *J Palliat Med.* 2013;16(6):645-652.
16. Rosenberg AR, Starks H, Jones B. "I know it when I see it." The complexities of measuring resilience among parents of children with cancer. *Support Care Cancer.* 2014.
17. Rosenberg A, Yi-Frazier JP, Wharton W, Crouch JD, Wolfe J, Syrjala KL, Baker KS. Developing Resilience Interventions for Adolescents and Young Adults with Cancer and their Families. Paper presented at: Kathleen Foley Palliative Care Research Retreat2013; San Francisco, CA.
18. Rosenberg A, Yi-Frazier JP, Wharton W, Crouch JD, Wolfe J, Syrjala KL, Baker KS Promoting Resilience in Adolescents and Young Adults with Cancer. Paper presented at: CureSearch for Children's Cancer Symposium2013; Washington, D.C.
19. Bradshaw BG, Richardson GE, Kumpfer K, et al. Determining the efficacy of a resiliency training approach in adults with type 2 diabetes. *Diabetes Educ.* 2007;33(4):650-659.
20. Lyon ME, Garvie PA, McCarter R, Briggs L, He J, D'Angelo LJ. Who will speak for me? Improving end-of-life decision-making for adolescents with HIV and their families. *Pediatrics.* 2009;123(2):e199-206.
21. Tan L, Martin G. Taming the Adolescent Mind: Preliminary report of a mindfulness-based psychological intervention for adolescents with clinical heterogeneous mental health diagnoses. *Clin Child Psychol Psychiatry.* 2012;Epub ahead of print.
22. Fjorback LO, Arendt M, Ornbøl E, Fink P, Walach H. Mindfulness-based stress reduction and mindfulness-based cognitive therapy: a systematic review of randomized controlled trials. *Acta Psychiatr Scand.* 2011;124:102-119.
23. Karlson CW, Rapoff MA. Attrition in randomized controlled trials for pediatric chronic conditions. *J Pediatr Psychol.* 2009;34:782-793.

24. Nansel TR, Iannotti RJ, Liu A. Clinic-integrated behavioral intervention for families of youth with type 1 diabetes: randomized clinical trial. *Pediatrics.* 2012;129(4):e866-873.
25. Connor KM, Davidson JR. Development of a new resilience scale: the Connor-Davidson Resilience Scale (CD-RISC). *Depress Anxiety.* 2003;18(2):76-82.
26. Snyder CR, Harris C, Anderson JR, et al. The will and the ways: development and validation of an individual-differences measure of hope. *J Pers Soc Psychol.* 1991;60(4):570-585.
27. Tomich PL, Helgeson VS, Nowak Vache EJ. Perceived growth and decline following breast cancer: a comparison to age-matched controls 5-years later. *Psychooncology.* 2005;14(12):1018-1029.
28. Kessler RC, Andrews G, Colpe LJ, et al. Short screening scales to monitor population prevalences and trends in non-specific psychological distress. *Psychol Med.* 2002;32(6):959-976.
29. Bjelland I, Dahl AA, Haug TT, Neckelmann D. The validity of the Hospital Anxiety and Depression Scale. An updated literature review. *J Psychosom Res.* 2002;52(2):69-77.
30. Varni JW, Burwinkle TM, Katz ER, Meeske K, Dickinson P. The PedsQL in pediatric cancer: reliability and validity of the Pediatric Quality of Life Inventory Generic Core Scales, Multidimensional Fatigue Scale, and Cancer Module. *Cancer.* 2002;94(7):2090-2106.
31. Association AM. Guidelines for Asolescents Preventative Services Monograph. 1997; http://www.ama-assn.org//resources/doc/ad-hlth/gapsmono.pdf. Accessed 08/01/2011.
32. Campbell-Sills L, Cohan SL, Stein MB. Relationship of resilience to personality, coping, and psychiatric symptoms in young adults. *Behav Res Ther.* 2006;44(4):585-599.
33. Connor KM, Vaishnavi S, Davidson JR, Sheehan DV, Sheehan KH. Perceived stress in anxiety disorders and the general population: a study of the Sheehan stress vulnerability scale. *Psychiatry Res.* 2007;151(3):249-254.
34. Davidson JR, Stein, D. J. Rothbaum, B.O., Pedersen, R., Tien, X.W., Musgnung, J. Resilience as a predictor of remission in post-traumatic stress disorder. . US Psychiatric and Mental Health Congress; 2006; New Orleands, LA.
35. Bruwer B, Emsley R, Kidd M, Lochner C, Seedat S. Psychometric properties of the Multidimensional Scale of Perceived Social Support in youth. *Compr Psychiatry.* 2008;49(2):195-201.
36. Irving LM, Snyder CR, Crowson JJ, Jr. Hope and coping with cancer by college women. *J Pers.* 1998;66(2):195-214.
37. Strauss A, Corbin, J. *Basics of qaulitative reserach techniques and procedures for developing grounded theory.* Thousand Oaks, CA: Sage; 1998.
38. Phipps S, Long AM, Ogden J. Benefit Finding Scale for Children: preliminary findings from a childhood cancer population. *J Pediatr Psychol.* 2007;32(10):1264-1271.
39. Tomich PL, Helgeson VS. Five years later: a cross-sectional comparison of breast cancer survivors with healthy women. *Psychooncology.* 2002;11(2):154-169.
40. Tomich PL, Helgeson VS. Is finding something good in the bad always good? Benefit finding among women with breast cancer. *Health Psychol.* 2004;23(1):16-23.
41. Kessler RC, Green JG, Gruber MJ, et al. Screening for serious mental illness in the general population with the K6 screening scale: results from the WHO World Mental Health (WMH) survey initiative. *Int J Methods Psychiatr Res.* 2010;19 Suppl 1:4-22.
42. Furukawa TA, Kessler RC, Slade T, Andrews G. The performance of the K6 and K10 screening scales for psychological distress in the Australian National Survey of Mental Health and Well-Being. *Psychol Med.* 2003;33(2):357-362.
43. Green JG, Gruber MJ, Sampson NA, Zaslavsky AM, Kessler RC. Improving the K6 short scale to predict serious emotional disturbance in adolescents in the USA. *Int J Methods Psychiatr Res.* 2010;19 Suppl 1:23-35.
44. Kessler RC, Barker PR, Colpe LJ, et al. Screening for serious mental illness in the general population. *Arch Gen Psychiatry.* 2003;60(2):184-189.
45. Rosenberg AR, Dussel V, Kang T, et al. Psychological Distress in Parents of Children With Advanced Cancer. *JAMA Pediatr.* 2013:1-7.
46. Verhoof EJ, Maurice-Stam H, Heymans HS, Evers AW, Grootenhuis MA. Psychosocial well-being in young adults with chronic illness since childhood: the role of illness cognitions. *Child Adolesc Psychiatry Ment Health.* 2014;8(1):12.
47. van der Geest IM, van Dorp W, Hop WC, et al. Emotional distress in 652 Dutch very long-term survivors of childhood cancer, using the hospital anxiety and depression scale (HADS). *J Pediatr Hematol Oncol.* 2013;35(7):525-529.

48. Varni JW, Limbers CA. The PedsQL 4.0 Generic Core Scales Young Adult Version: feasibility, reliability and validity in a university student population. *J Health Psychol.* 2009;14(4):611-622.
49. Hinds PS, Gattuso JS, Billups CA, et al. Aggressive treatment of non-metastatic osteosarcoma improves health-related quality of life in children and adolescents. *Eur J Cancer.* 2009;45(11):2007-2014.
50. Zebrack BJ, Ganz PA, Bernaards CA, Petersen L, Abraham L. Assessing the impact of cancer: development of a new instrument for long-term survivors. *Psychooncology.* 2006;15(5):407-421.
51. Husson O, Zebrack B. Psychometric Evaluation of an Adolescent and Young Adult (AYA) module of the Impact of Cancer Instrument,. *Journal of Adolescent and Young Adult Oncology.* in press.
52. Miller KA, Wojcik KY, Ramirez CN, et al. Supporting long-term follow-up of young adult survivors of childhood cancer: Correlates of healthcare self-efficacy. *Pediatr Blood Cancer.* 2016;epub ahead of print.
53. Economic Research Service U. U.S. Household Food Security Survey Module: Three-Stage Design, with Screeners. 2008; http://www.ers.usda.gov/briefing/foodsecurity/surveytools/hh2008.pdf. Accessed 3/19/2013.
54. Blumberg SJ, Bialostosky K, Hamilton WL, Briefel RR. The effectiveness of a short form of the Household Food Security Scale. *Am J Public Health.* 1999;89(8):1231-1234.
55. DA F. Children's HealthWatch Survey. 2010; http://www.childrenshealthwatch.org/upload/resource/englishinterview_revision4a.pdf. Accessed 3/19/13.
56. Nansel TR, Iannotti RJ, Liu A. Clinic-integrated behavioral intervention for families of youth with type 1 diabetes: randomized clinical trial. *Pediatrics.* 2012;124:e866-873.
57. Laffel LMB, Brackett J, Ho J, Ander BJ. Changing the process of diabetes care improves metabolic outcomes and reduces hospitalizations. *Quality Management in Health Care.* 1998;6:53-62.
58. Svoren BM, Butler D, Levine BS, Anderson BJ, Laffel LMB. Reducing acute adverse outcomes in youths with type 1 diabetes: A randomized, controlled trial. *Pediatrics.* 2003;112:914-922.
59. Raudenbush SW, Bryk AS. *Hierarchical linear models : applications and data analysis methods.* 2nd ed. Thousand Oaks: Sage Publications; 2002.
60. Lachin JM. Power and sample size evaluation for the Cochran-Mantel-Haenszel mean score (Wilcoxon rank sum) test and the Cochran-Armitage test for trend. *Stat Med.* 2011;30(25):3057-3066.
