# Supplementary material for: Assessment of the Promoting Resilience in Stress Management Intervention for Adolescent and Young Adult Survivors of Cancer at 2 Years: Secondary Analysis of a Randomized Clinical Trial
Source: JAMA Netw Open. 2021 Nov 24;4(11):e2136039. doi: 10.1001/jamanetworkopen.2021.36039 (PMC8613597; doi:10.1001/jamanetworkopen.2021.36039)
Supplement: Supplement 2. — eTable. Mean Participant-Reported Instrument Scores at Time of Enrollment, 6 Months, 12 Months, and 24 Months [file jamanetwopen-e2136039-s002.pdf]

Supplemental Online Content

Rosenberg AR, Zhou C, Bradford MC, et al. Assessment of the promoting resilience in stress management intervention for adolescent and young adult survivors of cancer at 2 years: secondary analysis of a randomized clinical trial. *JAMA Netw Open.* 2021;4(11):e2136039. doi:10.1001/jamanetworkopen.2021.36039

eTable. Mean Participant-Reported Instrument Scores at Time of Enrollment, 6 Months, 12 Months, and 24 Months

This supplemental material has been provided by the authors to give readers additional information about their work.

© 2021 Rosenberg AR et al. *JAMA Network Open.*

eTable. Mean Participant-Reported Instrument Scores at Time of Enrollment, 6 Months, 12 Months, and 24 Months

	Baseline		6-months		12-months		2-years	
**Well-Being Construct (Instrument)**	PRISM (n=48)	UC (n=44)	PRISM (n=36)	UC (n=38)	PRISM (n=34)	UC (n=33)	PRISM (n=31)	UC (n=26)
**Cancer-Related Quality of Life (PedsQL Cancer Module)**	65.5 (15.9)	65.2 (21.3)	72.3 (11.3)	63.8 (20.2)	74.2 (16.8)	68.5 (20.4)	74.2 (13.2)	70.2 (22.8)
**Hope (Hope Scale)**	48.7 (8.4)	50.7 (8.1)	51.5 (7.4)	50.0 (6.8)	51.3 (8.2)	47.5 (8.3)	51.1 (10.4)	49.8 (6.4)
**Resilience (10-item Connor-Davidson Resilience Scale)**	29.3 (6.2)	28.3 (5.8)	30.1 (5.2)	27.7 (6.2)	29.2 (7/3)	27.2 (7.0)	29.6 (7.2)	28.3 (6.9)
**Psychological Distress (Kessler-6 Psychological Distress Scale)**	6.1 (4.5)	7.6 (4.8)	5.1 (3.1)	7.1 (4.8)	5.6 (4.5)	6.5 (5.4)	5.6 (3.9)	6.3 (5.0)

Legend: All instrument scores reported as mean (standard deviation). PRISM = Promoting Resilience in Stress Management; UC = Usual Care

© 2021 Rosenberg AR et al. *JAMA Network Open.*
