# Supplementary material for: Assessment of the Promoting Resilience in Stress Management Intervention for Adolescent and Young Adult Survivors of Cancer at 2 Years: Secondary Analysis of a Randomized Clinical Trial
Source: JAMA Netw Open. 2021 Nov 24;4(11):e2136039. doi: 10.1001/jamanetworkopen.2021.36039 (PMC8613597; doi:10.1001/jamanetworkopen.2021.36039)
Supplement: Supplement 3. — Data Sharing Statement [file jamanetwopen-e2136039-s003.pdf]

Rosenberg AR, Zhou C, Bradford MC, et al. Assessment of the promoting resilience in stress management intervention for adolescent and young adult survivors of cancer at 2 years. *JAMA Netw Open.* 2021;4(11):e2136039. doi:10.1001/jamanetworkopen.2021.36039

Data Sharing Statement

Data

Data available: No

Additional Information

Explanation for why data not available: This study includes minor-aged patients; their parents did not provide consent for all details/data to be shared
